# Worry and insomnia as risk factors for depression during initial stages of COVID-19 pandemic in India

**DOI:** 10.1371/journal.pone.0243527

**Published:** 2020-12-10

**Authors:** Sahil Bajaj, Karina S. Blair, Amanda Schwartz, Matthew Dobbertin, R. James R. Blair

**Affiliations:** 1 Multimodal Clinical Neuroimaging Laboratory (MCNL), Center for Neurobehavioral Research, Boys Town National Research Hospital, Boys Town, NE, United States of America; 2 Program for Trauma and Anxiety (PTAC), Center for Neurobehavioral Research, Boys Town National Research Hospital, Boys Town, NE, United States of America; Charite Medical University Berlin, GERMANY

## Abstract

The biggest nationwide COVID-19 pandemic lockdown worldwide was enforced in India for an initial period of 21-days. Emerging evidence suggests that pandemic situations and associated lockdowns have an adverse impact on sleep and mental health. However, prediction of sleep health from sociodemographic characteristics and the public’s worry during the initial stages of the COVID-19 pandemic has not been extensively explored so far. It’s also unclear whether sleep outcomes mediate the association between worry and mental health during pandemic situations. A web-survey (N = 391) on sociodemographic characteristics, COVID-19 related worry, sleep health (insomnia and daytime sleepiness), and depression was conducted during the initial 21-days of the COVID-19 stringent lockdown in India. Multiple regression analyses showed that variables, including sex, age, income level, and worry score, contributed to the significant regression equation for insomnia but not for daytime sleepiness. Specifically, the female, younger, lower income, and highly worried populations contributed significantly more than the male, elderly, higher income, and less worried populations, respectively, to the prediction of insomnia. Mediation analyses showed that insomnia, but not daytime sleepiness, fully mediated the relationship between worry score and severity of depressive symptoms. We provide evidence that the female, younger, lower income, and worried populations may be at higher risk for insomnia during pandemic situations. Current evidence gives hope that improving sleep may reduce depressive symptoms during a pandemic situation. This underscores the importance of the implementation of effective public health policies in conjunction with strategical responses to the COVID-19 pandemic.

## Introduction

A cluster of cases of pneumonia led to the detection of the highly contagious, novel Corona Virus Disease 2019 (COVID-19) in December 2019. To slow the spread of this global pandemic, voluntary nationwide lockdowns were proposed and implemented around the globe. One of the biggest COVID-19 initial lockdowns was enforced in India where more than 1.3 billion people were strictly ordered to stay inside their homes for 21 days (25^th^ March 2020 to 14^th^ April 2020). Considerable work has focused on the pathophysiology of the disease and the further identifying the underlying biomarkers. But such a global pandemic event may also have severe impacts on sleep and mental health due to worries about the illness and associated economic and personal uncertainties. The current study aims to address these concerns and predict Indian residents’ sleep health from a variety of sociodemographic characteristics (i.e., sex, age, and income level) and the public’s worry during the COVID-19 situation, and further determine whether sleep health mediates the association between the individual’s worry towards the pandemic and their mental health, particularly depression.

Disrupted sleep has a disproportionate impact on immune system functioning [1, 2] and thus on the potential prevention, and/or treatment, of an infectious disease such as COVID-19. However, sleep parameters are influenced by a number of sociodemographic factors *including* sex, age, income status, and current personal worries. A meta-analysis study showed that females, particularly elderly females, are at greater risk of insomnia (i.e., either having difficulty in sleep maintenance or sleep onset) [3]. Females are also more likely to have greater daytime sleepiness (a measure of unintentional sleep at inappropriate times during the day) than males and experience a greater impact of excessive sleepiness on multiple activities of everyday living [4]. Age is also positively associated with insomnia [5, 6], whereas excessive daytime sleepiness has been reported to decrease with increasing age [7, 8]. Lower socioeconomic status is one of the most consistent predictors of both greater insomnia and excessive daytime sleepiness [9–12]. Worry, *including* trait worry [13] and COVID-19 related worry [14], has also been associated with insomnia. In addition, excessive daytime sleepiness has been indirectly linked to worry [15]. However, prior work suggested that other than poor sleep, factors such as psychological arousal or misperception about sleep need may contribute to daytime difficulties [16]. Therefore, the first purpose of the current study was to determine the extent to which sex, age, income level, and respondents’ worry predict both insomnia and daytime sleepiness in a population experiencing a particularly *stringent* COVID-19 lockdown.

The COVID-19 outbreak has also had a moderate to severe impact on mental health, including increasing depressive symptomatology [17], a particular concern as the mental impact of an epidemic may outlast the disease itself [18]. Both worry about stressful events and insomnia are associated with poor mental health, including depressive symptomology [19–22]. There are data indicating that the relationship between stressful events and mental health is stronger for individuals with poor sleep health as compared to individuals with better sleep quality [23, 24]. Insomnia may disrupt appropriate emotion reactivity and emotion regulation increasing the risk for depression [25]. The mediation of the association between worry and depression by sleep health may be particularly exaggerated during the COVID-19 outbreak because of worries relating to the pandemic itself. The second purpose of the current study was to examine the extent to which individuals’ sleep health mediates the association between COVID-19 related worry scores and severity of depressive symptoms.

The current study focused on an Indian sample population during the initial 21-days of stringent and largest COVID-19 lockdown. Based on prior published work, we hypothesized greater insomnia and excessive daytime sleepiness in the female, elderly, lower income, and highly worried populations relative to the male, younger, higher income, and less worried populations. Second, we hypothesized that both insomnia and daytime sleepiness would mediate the association between worry score during COVID-19 pandemic and severity of depressive symptoms.

## Materials and methods

### Study design

The initial COVID-19 nationwide lockdown in India was ordered on 24^th^ March 2020 for 21-days and began on 25^th^ March 2020. Our aim was to take advantage of this unique opportunity and collect data from a sample during a period when worry was acute and people were not acclimated to the lockdown conditions. To accomplish this, we used freely-available Google Forms software to create an online survey about sociodemographic characteristics, COVID-19 related worry score, sleep health, and severity of depressive symptoms and obtained the expedited IRB approval within the first 8 days (25^th^ March 2020-1^st^ April 2020) of this 21-day lockdown period (25^th^ March 2020-14^th^ April 2020). Data were subsequently collected between April 2^nd^ and April 14^th^, 2020—i.e., during 2^nd^ and 3^rd^ week of initial lockdown. Respondents were explicitly asked to score their worry levels, sleep quality (i.e., insomnia and daytime sleepiness) and severity of depressive symptoms that were present specifically during this initial 21-day lockdown due to COVID-19. The details about the study and the survey link were openly shared on several social media platforms, including LinkedIn, Twitter, and Facebook groups, targeting current Indian nationals residing in India. An online consent was obtained from the participants. Participation was completely voluntary, and participants were not paid for their time. The study was approved by the IRB at Boys Town National Research Hospital.

### Participants

A total of 408 participants completed the survey. Participants were explicitly asked about their age group and city/state of residence during the 21-day COVID-19 lockdown to determine their eligibility. Any current Indian resident aged 19 years or older was eligible for the study. Data from a total of 17 participants, who either responded to the survey from outside India or did not answer all the questions, were excluded from the analysis.

### Data acquisition

#### Measures

The main outcome measures were answers to the Sociodemographic Questionnaire, the COVID-19 related Worry Questionnaire, the Sleep Health Questionnaire, and the Mood and Feelings Questionnaire as following:

*Sociodemographic Questionnaire*. Data related to sociodemographic characteristics included sex, age (indexed via age band: 18–23, 24–29, 30–35, 36–41, or older than 41 years), and monthly family income in rupees (<20,000, 20,000–40,000, 40,000–80,000, >80,000). Because of limited use of social media platforms in the elderly population, data were not sub-categorized above the age of 41 years.

*COVID-19 Worry Questionnaire*. The Worry Questionnaire was developed to assess participants’ worry scores with respect to the COVID-19 pandemic. Respondents were asked to indicate their level of agreement on a 5-point Likert Scale where strongly disagree = 1 and strongly agree = 5 on 8 statements/questions related to their state of mind during COVID-19 lockdown (Table 1). The total score ranged between 8 and 40. In the current sample, the COVID-19 Worry Scale showed moderate internal consistency (Cronbach’s alpha = .68). A Cronbach’s alpha (α) between .6 and .7 indicates an acceptable level of reliability [26]. Also, a Cronbach’s alpha of .5 is common for scales consisting of less than 10 items [27].

**Table 1:** Questions/statements included in COVID-19 Worry Questionnaire.

**1**	I do not care whatsoever about the CORONA virus (or lockdown) (reverse scaled)
**2**	I think a lot about what will happen to the world
**3**	I really worry and want this to end as soon as possible
**4**	I thought about my health
**5**	I thought about others
**6**	Did you try to look at a situation as if you were the other person who is infected by Corona?
**7**	I thought about myself
**8**	I thought about the future

*Sleep Health Questionnaire*. The following standard insomnia and daytime sleepiness scales were used to quantify the sleep health of the respondents:

*Insomnia Severity Index (ISI)* is a brief self-administered seven-item questionnaire that assesses perception of both nocturnal and diurnal symptoms of insomnia [28]. The total ISI score ranged between 0 and 28 with 0–7 a prior threshold indicating no clinically significant insomnia, 8–14 indicating subthreshold insomnia, 15–21 indicating clinical insomnia with moderate severity, and 22–28 indicating clinical insomnia with severe severity. The scale has shown excellent test-retest reliability (intra-class correlation coefficient of .84) [29] and internal consistency for both community (Cronbach α = .90) and clinical insomnia (Cronbach α = .91) samples [30].

*Epworth Sleepiness Scale (ESS)* is a brief self-administered eight-item questionnaire that assesses general levels of daytime sleepiness [31]. The total ESS score ranged between 0 and 24 with 0–5 a priori threshold indicating lower normal daytime sleepiness, 6–10 indicating higher normal daytime sleepiness, 11–12 indicating mild excessive daytime sleepiness, 13–15 indicating moderate excessive daytime sleepiness, and 16–24 indicating severe excessive daytime sleepiness. The scale has shown a high level of internal consistency (Cronbach α = .88) [32]. The scale has also been well-validated in terms of its high divergent validity and reproducibility in both healthy subjects and clinical sleep patients [31, 33, 34].

*Mood and Feelings Questionnaire (MFQ)*. The MFQ is a self-administered 33-item questionnaire developed to assess severity of depressive symptoms [35, 36]. The MFQ has demonstrated satisfactory validity and internal consistency (Cronbach α = .91 to .93) [37]. Due to time constraints, a slightly shorter version of the MFQ was administered consisting of 25 of the 33 items (“I felt I was no good anymore,” “I blamed myself for things that weren’t my fault,” “I thought my family would be better off without me,” “I hated myself,” “I felt I was a bad person,” “I worried about aches and pains,” “I thought nobody really loved me,” and “I did everything wrong” were excluded).

### Data analysis

#### Multiple regression analysis

A multiple regression basically predicts dependent variables from continuous independent variables. Including categorical independent variables as binary/dummy variables in the regression analysis may accurately predict the dependent variables [38]. The current data hypothesize that the categorical independent variables (i.e., sex, age levels, income levels, and worry levels) may affect the variance in the dependent variables (i.e., insomnia and daytime sleepiness). Therefore, the collected data (N = 391, 209 females) were divided into (i) four levels based on age (Age Level 1: age range 19–29 years, N = 169, 94 females; Age Level 2: age range 30–35 years, N = 107, 62 females; Age Level 3: age range 36–41 years, N = 60, 30 females; and Age Level 4: age > 41 years, N = 55, 23 females), (ii) two levels based on family income (Income Level 1: income < 40,000 INR, N = 138, 76 females and Income Level 2: income ≥ 40,000 INR, N = 253, 133 females), and (iii) two levels based on magnitude of worry scores (Worry Level 1: worry score ≤ 33, N = 230, 125 females and Worry Level 2: worry score > 33, N = 161, 84 females). Because of psychological developmental differences, the population aged between 18 and 29 years (Age Level 1), also considered as “emerging adulthood” [39], was chosen as the “reference” level against which the other age levels were compared. India’s per capita net national income was reported to be about 10,057 INR during 2017–2019 [40]. The average household size from the current data was 3.87±2.18, therefore, the threshold for income level (< INR 40,000) was calculated based on the average household income of four adults. The threshold for worry score (> 33) for highly worried individuals was based on the median value (= 33) of worry score.

Females, income level 1, and worry level 1 were each coded as "0," whereas males, income level 2, and worry level 2 were each coded as "1." The categorical variables for age levels were converted to dummy variables by excluding one of the age levels (i.e., age level A1 as the reference level). A multiple regression analysis using "enter" method was performed to determine to what extent the independent variables converted to binary and dummy variables predicted the dependent variables (insomnia and daytime sleepiness). Here, contribution of each of the independent variables (relative to the reference levels) to the variance when predicting insomnia and daytime sleepiness were also calculated.

#### Mediation analysis

A standard mediation analysis (bootstrap samples = 10,000) was conducted using the Hayes PROCESS program (version 3.5) (https://www.processmacro.org/download.html) in SPSS (version 25) to determine the significance of indirect effects (95% confidence intervals), i.e., to determine the extent at which insomnia and daytime sleepiness individually mediate the association between magnitude of worry scores and severity of depressive symptoms.

## Results

Table 2 summarizes sociodemographic characteristics of interest and measures of COVID-19 related worry score, sleep (insomnia and daytime sleepiness), and severity of depressive symptoms. 53.45% and 19.69% of the participants reported low to severe levels of insomnia (i.e., ISI ≥8) and daytime sleepiness (i.e., ESS ≥11), respectively (levels notably higher than those reported in typical samples not exposed to severe situational stress; 18.6% & 14% respectively [41, 42]).

**Table 2:** Sociodemographic characteristics and measures of worry score, sleep, and severity of depressive symptoms.

***Sociodemographic Characteristics (% of participants)***	
Sex	Females (53.45)
	Males (46.55)
Age Levels in Years	Level 1: 19–29 (43.22)
	Level 2: 30–35 (27.37)
	Level 3: 36–41 (15.35)
	Level 4: > 41 (14.07)
Income Levels	Level 1: Low Income i.e., < 40,000 (35.29)
	Level 2: High Income i.e., ≥ 40,000 (64.71)
Worry Levels	Level 1: Low Worry ≤ 33 (58.82)
	Level 2: High Worry > 33 (41.18)
***Outcomes (mean ± standard deviation)***	
Worry Score	32.68 ± 3.74
Insomnia	8.59 ± 5.79
Daytime Sleepiness	7.35 ± 3.93
Depression	11.63 ± 9.23

### Prediction of insomnia and daytime sleepiness from sociodemographic characteristics and worry scores

Multiple regressions were run to predict insomnia and daytime sleepiness from sex, age, income level, and the worry score as independent categorical (binary/dummy) variables. There were significant negative associations of insomnia with sex, age levels 3 and 4, and income level, and a significant positive association between insomnia and worry level (Table 3). Insomnia revealed a significant regression equation (F (6,384) = 6.53, *p <* .*001*, R^2^ = .09) indicating that sex, age, income level, and worry scores significantly predicted insomnia. There were significantly greater contributions from females than males (B = -1.51, *p =* .*008*), from the relatively younger population (Age Level A1) than the elderly populations (Age Level A3: B = -2.14, *p =* .*01* and Age Level A4: B = -2.31, *p =* .*01*), from the lower income population (Income Level 1) than the higher income population (Income Level 2) (B = -1.28, *p =* .*04*), and from the highly worried population (Worry Level 2) than the less worried population (Worry Level 1) (B = 1.66, *p =* .*004*). These findings are summarized in Table 4. Daytime sleepiness did not reveal a significant regression equation (F (6,384) = .67, *p =* .*67*, R^2^ = .01)). It is worth noting here a follow-up multiple regression revealed that the demographic variables significantly predicted worry scores (F (5,385) = 2.71, *p =* .*02*, R^2^ = .03). Specifically, there was greater contribution from the relatively younger population (Age Level A1) than the elderly populations (Age Level A3: B = -1.06, *p =* .*06* (i.e., at the trend level) and Age Level A4: B = -2.09, *p <* .*001*).

**Table 3:** Pearson correlations among the variables of interest (*p < 0.*05*).

	ISI	Sex	Age Level 2	Age Level 3	Age Level 4	Income Level	Worry Level
**ISI**	-						
**Sex**	-0.143*	-					
**Age Level 2**	0.001	-0.055	-				
**Age Level 3**	-0.118*	0.029	-0.261*	-			
**Age Level 4**	-0.140*	0.094	-0.248*	-0.172*	-		
**Income Level**	-0.154*	0.024	0.069	0.121*	0.160*	-	
**Worry Level**	0.160*	0.021	-0.001	-0.068	-0.084	-0.013	-

**Table 4:** Multiple regression analysis on how categorical variables predict insomnia (*p < 0.*05*).

Model 1	Unstandardized B	Standard Error	Standardized Coefficients β	t	Significance	Variance Inflation Factor (VIF)
**(Constant)**	10.328	0.638	-	16.186	0.000	-
**Sex**	-1.513	0.568	-0.130	-2.665	0.008*	1.013
**Age Level 2**	-0.879	0.699	-0.068	-1.258	0.209	1.227
**Age Level 3**	-2.139	0.858	-0.133	-2.492	0.013*	1.209
**Age Level 4**	-2.309	0.897	-0.139	-2.575	0.010*	1.228
**Income Level**	-1.279	0.613	-0.106	-2.087	0.038*	1.084
**Worry Level**	1.659	0.577	0.141	2.876	0.004*	1.019

### Mediation analysis: Role of insomnia and daytime sleepiness in mediating the association between worry score and depression

To test the hypothesis that both insomnia and daytime sleepiness individually mediate the relationship between worry and depression, standard mediation analyses were conducted.

#### Insomnia

**Fig 1:**
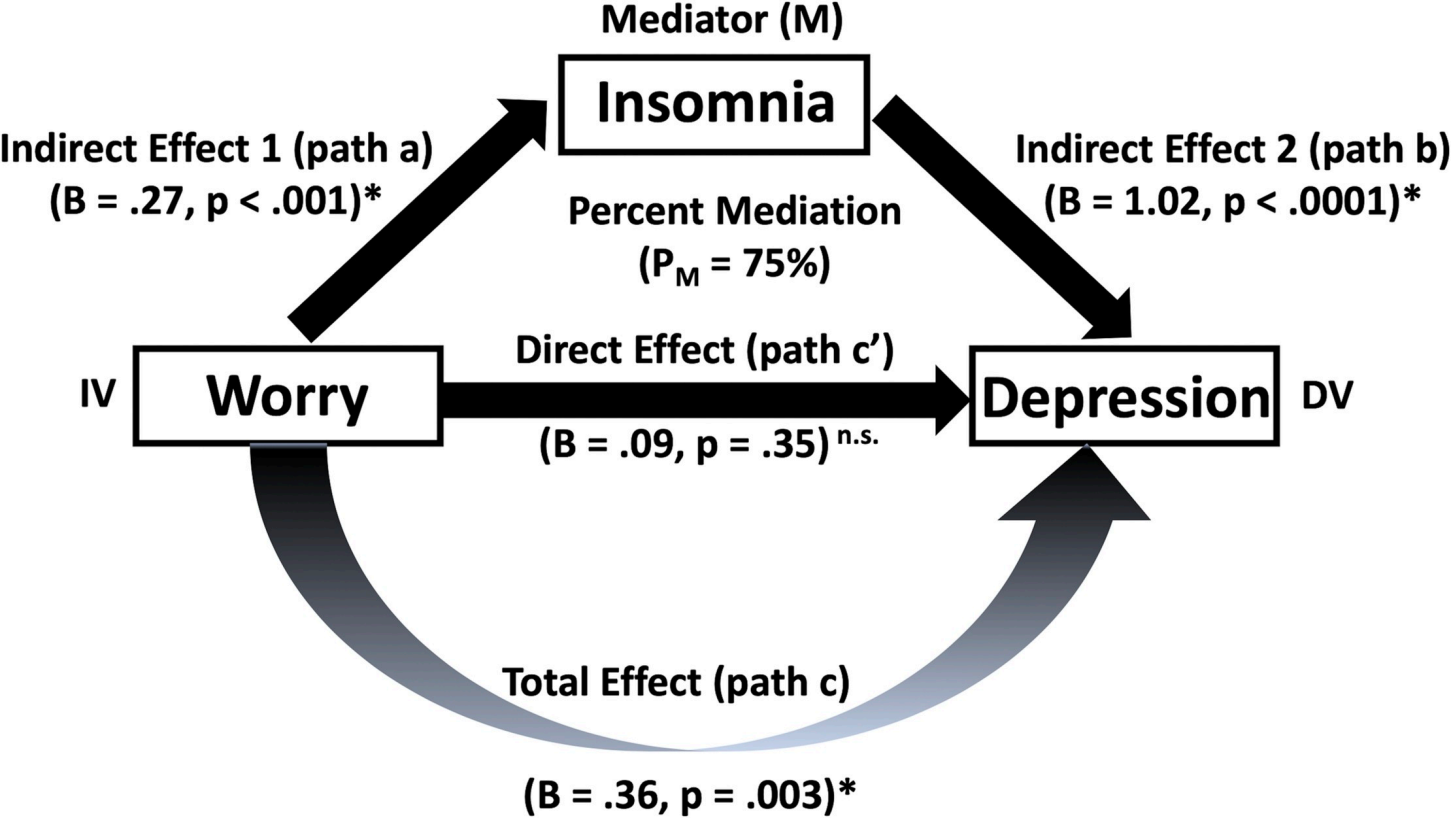
Mediation analysis. An illustration of the path model (model 4) used to test the mediation effect of insomnia (M) on the association of worry score (independent variable, IV) with depression (dependent variable, DV). Path model shows complete mediation effect of insomnia, with percent mediation (P_M_) of 75%. B, unstandardized beta coefficients; *p < .*005*.

Greater worries over the COVID-19 pandemic were significantly associated with both more self-reported insomnia (r = .17, *p =* .*001*) and increased severity of depressive symptoms (r = .15, *p =* .*003*). Insomnia severity was also (independent of worry score) correlated with severity of depressive symptoms (r = .64, *p <* .*001*). A standard mediation analysis revealed that while worries over the COVID-19 pandemic were associated with increased severity of depressive symptoms (total effect, c = .36, *p =* .*003*), this association completely disappeared once insomnia was included as an "intervening" factor (direct effect, c’ = .09, *p =* .*35*). The bootstrap confidence interval for the indirect effect (ab = .27; [.11 .44] 95% CI) did not include zero. The percent mediation (P_M_) (i.e., percent of the total effect (c) accounted for by indirect effect (ab)) was 75%. Findings indicate that insomnia completely accounted for the association between worries and severity of depressive symptoms over the COVID-19 pandemic. Fig 1 illustrates the path model (model 4) used to test the mediation effect of insomnia on the association of worry score with depression.

#### Daytime sleepiness

Greater worries over the COVID-19 pandemic were not significantly associated with greater self-reported daytime sleepiness (r = .08, *p =* .*12*). Therefore, follow-up mediation analysis was not conducted.

## Discussion

This study showed that insomnia, but not daytime sleepiness, can be significantly predicted from sex, age, income levels, and worry score. In addition, insomnia, but not daytime sleepiness, significantly mediated the relationship between COVID-19 related worry scores and severity of depressive symptoms. Below we discuss these findings in detail.

### Prediction of insomnia from sociodemographic characteristics and worry score

In accordance with our a priori hypotheses, females and individuals with lower incomes contributed greater than males and individuals with higher incomes in predicting insomnia. Contrary to our a priori hypotheses, the younger population group contributed greater than the elderly population group in predicting insomnia.

In line with the results of this study, previous work has indicated that females are usually at greater risk for insomnia than males [3]. Potentially, this relates to putative higher stress responsivity in females [43, 44]. Similarly, previous work showed that factors such as feelings of shame or hopelessness in conjunction with poor financial conditions, and strong association between poor socio-economic status and mental health problems may be linked with greater insomnia [9, 10, 45, 46]. This is consistent with the current findings of a positive association between lower income and insomnia. Moreover, the possibility of lower family income status could be one of the direct consequences of “unexpected” lower income caused by the pandemic lockdown, which might be in line with poor health associated with undesired, involuntary, and unexpected unemployment [47].

In contrast to predictions, however, younger individuals in the current study were more associated with insomnia than older individuals. In the previous literature, age is more typically found to be positively associated with insomnia [5, 6]. However, it should be noted that much of the literature related insomnia to higher ages [48–51] than the older bracket in the current study (>41 years). As such, previous studies may have missed a potential increased risk for insomnia in young adults relative to middle-aged adults. It is possible that due to limited life exposure and experience to daily life challenges, the young adults may have been at greater risk of experiencing stress during stressful times than the middle-aged adults. Certainly, it is notable that our multiple regression focused on predicting current worry from the demographic variables indicated that young adulthood was a greater contributor to worry than older adulthood.

In line with our predictions, the worried population during the COVID-19 pandemic contributed greater than the less worried population in predicting insomnia. This finding is consistent with prior evidence on insomnia and both COVID-19 specific worry [52] as well as worries during non-COVID-19 specific stressful situations such as a stressful work environment [53–55] and stress from family life [56]. In particular, worry is one of the strongest predictors of sleep disturbance [57]. Previous work has indicated that worrisome thoughts, including the repetitive thoughts, negative overthinking, cognitive arousal, and intrusive thoughts, have a major role to play in causing insomnia, difficulty falling asleep, poor sleep quality, and shorter sleep duration [58]. Indeed, the cognitive model of insomnia also suggests that negatively toned cognitive activity, which comprises worry and rumination, may be associated with insomnia [59]. Moreover, worrying about stressful events may lead to hyperarousal which is a central underlying driver of insomnia [60].

### Insomnia as a significant mediator for the association between worry score and depression

Worry has been associated with depression [61, 62]. Our secondary goal in this study was to determine the extent to which insomnia might mediate this association. As noted above, worry was significantly associated with insomnia in the current study, a finding in line with previous work [52, 57]. Critically, healthy sleep levels are important for healthy emotional processing. Disrupted sleep (e.g., insomnia) has been associated with emotional dysfunction and depressive symptomatology [21, 22, 63–65]. In line with our hypothesis, we found that significant association between COVID-19 related worry scores and severity of depressive symptoms was fully accounted for by insomnia. This indicates that greater levels of worry about the disease leads to difficulty in sleeping, which in turns leads to greater levels of depressive symptoms. Indeed, sleep problems may also cause a detrimental impact on neurotransmitter receptor function, alter regulation of the hypothalamic-pituitary-adrenal axis, and elevate the levels of stress hormones (cortisol and adrenaline), which may result in impaired thinking, emotional dysregulation, and stress related disorders such as depression [66–70]. Moreover, sleep disorders may also reduce the body’s affective response to effectively respond to stressors [71, 72].

### Role of daytime sleepiness

Contrary to our a priori hypotheses, but partially consistent with prior work, the data did not show strong associations of excessive daytime sleepiness with sex [8, 73–75], age [76, 77], socio-economic status [78, 79], or stressful situations [80, 81]. Also, we did not find daytime sleepiness to be a significant mediator for the relationship between worry score and severity of depressive symptoms. It is possible that the absence of a significant role of daytime sleepiness in the current study reflects the fact that the mean severity of daytime sleepiness for the current sample was consistent with most participants reporting no to slight chances of daytime sleepiness. Indeed, only 19.7% of the study sample reported an ESS score of greater than 10 which is an indicative of excessive daytime sleepiness.

## Limitations

First, the current data did not reflect whether lower family income was a direct consequence of the pandemic lockdown. Second, and relatedly, it could be argued that the participants’ insomnia levels might represent pre-existing conditions. While this cannot be discounted, it is worth noting that the rate for low to severe insomnia reported here was 53.45%. The rate typically observed in typical samples not exposed to severe situational stress is markedly lower (18.6%) [41]. As such, we believe that this markedly high rate of insomnia reflected a reaction to the lockdown. Third, given the total population of the country, our sample size was relatively small. This is because the data were strictly collected within the narrow timeframe of two weeks within the initial 21-day stringent lockdown. Fourth, this study completely relied on a web-based survey shared on social media platforms. This technique for data collection slightly over-sampled younger (70.59% aged 35 years or less whereas 65% of country’s population is below the age of 35) and affluent respondents (64.71% with monthly income 40,000 INR or more whereas the per capita net national income in the country was 10,057 INR per month during 2017–2019 [40]). Therefore, caution should be exercised when drawing conclusions when generalizing from the current results beyond relatively young and affluent participants. Fifth, some of the collected data were based on a newly introduced unvalidated self-reported measure. Therefore, again current findings should be interpreted with some caution. Future work should establish the reliability of newly introduced self-reported measures in this context. Future studies should also focus on the impact of time-varying (continuous data points) and longitudinal components (pre- and post-pandemic lockdown) on interview-based characteristics of sleep and depression across a larger sample size.

## Conclusions

Greater levels of insomnia were observed for the female, younger, lower income, and highly worried populations during the COVID-19 pandemic lockdown. Insomnia was found as a complete mediator in predicting severity of depressive symptoms from worry score. Current evidence gives hope and provides an effective pathway to overcome depression by treating insomnia. Therefore, to prevent and reduce the severity of depressive symptoms, even among the general population who are not typically affected by the COVID-19, there is a crucial need to pay attention to a nation’s sleep and mental health. It is extremely important for policy makers, health care professionals, and governments to implement effective public health policies in conjunction with strategical responses to pandemics. This will also help the scientific community to better understand the mental impact of the disease and effectively implement coping strategies in both prevention and treatment efforts of mental illness.
